# Principles and essential elements of the fundamental right to health in Colombia: an integrative review of structural determinants and systemic challenges

**DOI:** 10.3389/frhs.2026.1838181

**Published:** 2026-07-02

**Authors:** Edwin Mauricio Millan-Hernandez, Yamileth Ortiz-Gomez, James Frank Trujillo-Perdomo, Fabian Felipe Fernandez-Daza

**Affiliations:** 1 Facultad de Educación a Distancia y Virtual, Grupo de Investigación GISAP, Institución Universitaria Antonio José Camacho, Cali, Colombia; 2 Pontificia Universidad Javeriana, Instituto de Salud Pública, Bogotá, Colombia; 3 Institución Universitaria Antonio José Camacho, Cali, Colombia; 4 Facultad de Ciencias Básicas, Grupo de Investigación GIMIA, Universidad Santiago de Cali, Cali, Colombia

**Keywords:** health policies, health policy, health services research, legislation as topic, principles, values

## Abstract

**Objective:**

This study aims to describe the implementation of the principles and essential elements of the fundamental right to health (FRH) in Colombia.

**Methods:**

An integrative review was conducted between February 23 and March 6, 2021, using six databases. A total of 63 studies addressing the principles and elements of the FRH in Colombia were included.

**Results:**

Four higher-order analytical categories were identified: (1) barriers to access and responsiveness to health needs; (2) ethics, humanization of care, respect, and confidentiality; (3) insurance affiliation within the General System of Social Security in Health (SGSSS); and (4) availability, networks, and integration of healthcare services. These categories reflect structural, institutional, and relational dimensions shaping the realization of the FRH.

**Conclusions:**

The realization of the FRH in Colombia remains constrained by structural determinants, governance weaknesses, and systemic fragmentation, particularly affecting vulnerable populations. These findings highlight the need for integrated policy and governance reforms.

## Introduction

The General System of Social Security in Health (SGSSS, for its Spanish acronym) is defined as a comprehensive framework of institutions, regulations, and procedures designed to promote individual well-being and social integration, while ensuring quality of life and providing coverage for contingencies that adversely affect health and economic capacity (1).

In Colombia, the SGSSS is primarily governed by Law 100 of 1993 (1), Law 1438 of 2011 (2), and Law 1751 of 2015 (3). The latter, which constitutes the statutory health law, recognizes health as a fundamental right (FRH) and establishes a framework based on core principles and essential elements, including availability, acceptability, accessibility, quality, and professional competence. In addition, the law enshrines 14 guiding principles, such as universality, the pro homine principle, equity, continuity, timeliness, the primacy and progressivity of rights, free choice, sustainability, solidarity, efficiency, interculturality, and the protection of Indigenous, ROM, Black, Afro-Colombian, Raizal, and Palenquero communities.

Within this framework, y Soto Domínguez and Calderón Ossa (4) argue that achieving the objectives of the SGSSS requires the integrated application of principles such as universality, solidarity, equality, sufficiency, participation, and transparency. To improve the performance and responsiveness of the health system, Colombia has implemented a series of regulatory reforms, particularly in relation to financing mechanisms. Prior to Law No. 100 of 1993 (5), the system relied on contributions from employers (4.67%) and formal workers (2.33%), complemented by public subsidies for the support of public hospitals. However, limited coverage among non-contributing populations resulted in high out-of-pocket expenditures, reaching approximately 38% of total health spending.

With the enactment of Law No. 100 and its objective of achieving universal coverage by 2000, contributions from employers (8%) and formal workers (4%) were increased, alongside expanded fiscal resources in accordance with Law No. 60 of 1993. These funds were intended to finance the subsidized regime through a per capita payment unit (UPC), administered by health insurance entities (EPS) and transferred via the Solidarity and Guarantee Fund (FOSYGA).

Subsequent reforms further strengthened financing mechanisms. Law No. 1122 of 2007 increased total contributions to 12.5%, allocating additional resources to the subsidized regime. In 2010, fiscal efforts were expanded through taxes on tobacco, alcohol, and lotteries to address persistent gaps in coverage and equity. Later, Law No. 1607 of 2012 introduced a shift toward tax-based financing, reducing employer contributions and compensating the system through the income tax for equity (CREE). More recently, Law No. 1955 of 2019 allocated additional resources from value-added tax (VAT) (IVA, for its Spanish acronym) revenues and increased selective consumption taxes (5).

Overall, these reforms reflect a gradual transition toward a predominantly publicly financed health system (5). However, despite increased financial resources within the SGSSS, the effective realization of the fundamental right to health remains constrained, particularly in ensuring equitable access for vulnerable populations such as older adults and individuals with disabilities.

Furthermore, the enactment of Law No. 1438 of 2011 marked a reform of the SGSSS in Colombia, positioning primary healthcare (PHC) as a central strategy to strengthen a patient-, family-, and community-centered approach, thereby improving both the quality of care and access to health services.

Additionally, the National Development Plan 2014–2018 established the strategic framework for the Comprehensive Health Care Policy (CHCP) (PAIS, for its Spanish acronym) (6). These policies are grounded in the principles of primary healthcare and emphasize comprehensive risk management, as well as differentiated approaches tailored to diverse populations and territorial contexts (6, 7).

As part of its operationalization, the CHCP adopted the Integrated Health Care Model (MIAS, for its Spanish acronym), designed to ensure the timeliness, continuity, comprehensiveness, acceptability, and quality of healthcare services under conditions of equity. The MIAS comprises a structured set of prioritization processes, interventions, and institutional arrangements that guide the actions of health system stakeholders within a people-centered approach (7).

However, due to challenges encountered during its implementation, the Ministry of Health and Social Protection introduced the Territorial Integrated Action Model (MAITE, for its Spanish acronym) as a strategic adjustment aimed at strengthening territorial PHC governance and enhancing system performance (8).

Nevertheless, Colombia is currently engaged in an ongoing process of reflection and debate regarding a new proposal for healthcare system reform. This process is partly informed by the 1,719,584 tutela (guardianship) claims filed between 2008 and 2019. Of these, 404,046 claims filed in 2019 resulted in judicial decisions upholding the right to health following the enactment of the statutory law (9).

To date, several aspects related to key principles and essential elements—such as universality, accessibility, timeliness, quality, and professional competence—remain inadequately fulfilled (10). This situation suggests persistent challenges in the realization of the right to health, despite the enactment of the Statutory Health Law in 2015.

From a theoretical perspective, these challenges may be associated with weaknesses in health system governance, which can be understood as a structural factor shaping policy development, institutional arrangements, resource allocation, and accountability mechanisms within healthcare systems (11). Such governance limitations, in turn, influence the social determinants of health (12) and the conditions necessary to achieve the highest attainable standard of health from a rights-based perspective (13).

In alignment with this framework, this review examines the implementation of the fundamental right to health by identifying reported violations across empirical studies. By integrating these findings within a governance and rights-based perspective, the study provides a critical understanding of the structural drivers of health inequities and informs ongoing debates on the effective realization of the right to health. Accordingly, the study aims to describe the implementation of the principles and essential elements of the FRH through a systematic documentary analysis.

## Materials and methods

An integrative review (IR), defined as a methodological approach that synthesizes both theoretical and empirical literature to enhance the understanding of a specific phenomenon or problem, was conducted (14). This approach enables the inclusion of diverse study designs, encompassing both experimental and non-experimental studies.

For this review, a comprehensive search was performed across six electronic databases to identify studies addressing key concepts, theoretical perspectives, and complex issues related to healthcare. This process provided the foundation for the development of conceptual categories associated with the principles and essential elements of the FRH (3).

The objective of this review was to examine the discourse presented in the findings of selected studies and to extract empirical insights into the implementation of the FRH and its relationship with the 14 guiding principles and four essential elements established in Law No. 1751 of 2015 (3).

Search Strategy. A comprehensive literature search was conducted across six electronic databases, including Scopus, SciELO, LILACS, PubMed, ScienceDirect, and Web of Science. The search strategy combined keywords related to health policy, principles, and Colombia using Boolean operators (“AND”, “OR”), and applied database-specific filters such as publication year, language, and open access availability.

Searches were restricted to studies published between 2015 and 2021 in English or Spanish. This timeframe was selected to examine the evolution of the principles and essential elements associated with the realization of the fundamental right to health (FRH) in Colombia, using the implementation of Statutory Law No. 1751 of 2015 as a key reference point.

This period enables a consistent analysis of structural challenges, including barriers to access, fragmentation of service delivery, and inequalities associated with insurance coverage, ethnicity, and geographic location. It also allows for the examination of persistent issues, such as limited access to medicines, discontinuities in care, and concerns regarding the financial sustainability of the health system—challenges that predate the enactment of the law.

Although not the primary focus of this study, the selected timeframe also encompasses the COVID-19 pandemic (2020–2021), an exogenous shock that provides additional insight into the health system's capacity to respond to increased demand and operational constraints.

From a methodological perspective, this integrative review represents the initial phase of a broader doctoral research project aimed at identifying key analytical variables to inform subsequent empirical research in rural settings. Accordingly, the defined timeframe supports the development of conceptually grounded categories by ensuring that the evidence analyzed reflects the policy, institutional, and implementation conditions associated with the FRH under a consistent regulatory framework.

To ensure consistency and reproducibility, equivalent search strings were systematically adapted to the syntax and indexing structures of each database. This approach facilitated a comprehensive and comparable retrieval of evidence across sources while maintaining methodological rigor and transparency. The detailed search strategy is presented in Table 1.

**Table 1:** Search strategy.

#	Database	Search strategy	Date range	Results (*n*)
1	SciELO	politica AND salud AND colombia AND year_cluster (2015–2020) AND la:(es OR en)	2015–2020	177
2	Scopus	politica AND salud AND colombia AND filters (OA, country, year, language)	2015–2021	622
3	LILACS	(políticas) AND (salud) AND (colombia) AND filters (year, language)	2016–2021	504
4	ScienceDirect	politica AND salud AND colombia	2015–2021	645
5	SciELO	salud AND (principios OR elementos OR valores) AND colombia	2015–2020	114
6	ScienceDirect	salud AND (principios OR elementos OR valores) AND colombia	2015–2021	6000
7	Scopus	TITLE-ABS-KEY (health AND principles OR elements OR values AND colombia)	2015–2020	57
8	LILACS	salud AND (principios OR elementos OR valores) AND colombia	2015–2021	88
9	PubMed	políticas AND salud AND colombia	2015–2021	41
10	PubMed	(salud OR principios OR elementos OR valores) AND colombia	2015–2021	3624
11	Web of Science	politica AND salud AND colombia	2015–2021	4
12	Web of Science	salud AND (principios OR elementos OR valores) AND colombia	2015–2021	489

Total records identified: *n* = 12,365.

The search strategies, including the procedures applied, search queries, dates of retrieval, number of records identified per database, and corresponding links, are detailed in Supplementary Material 1 (Search Strategy).

**Figure 1:**
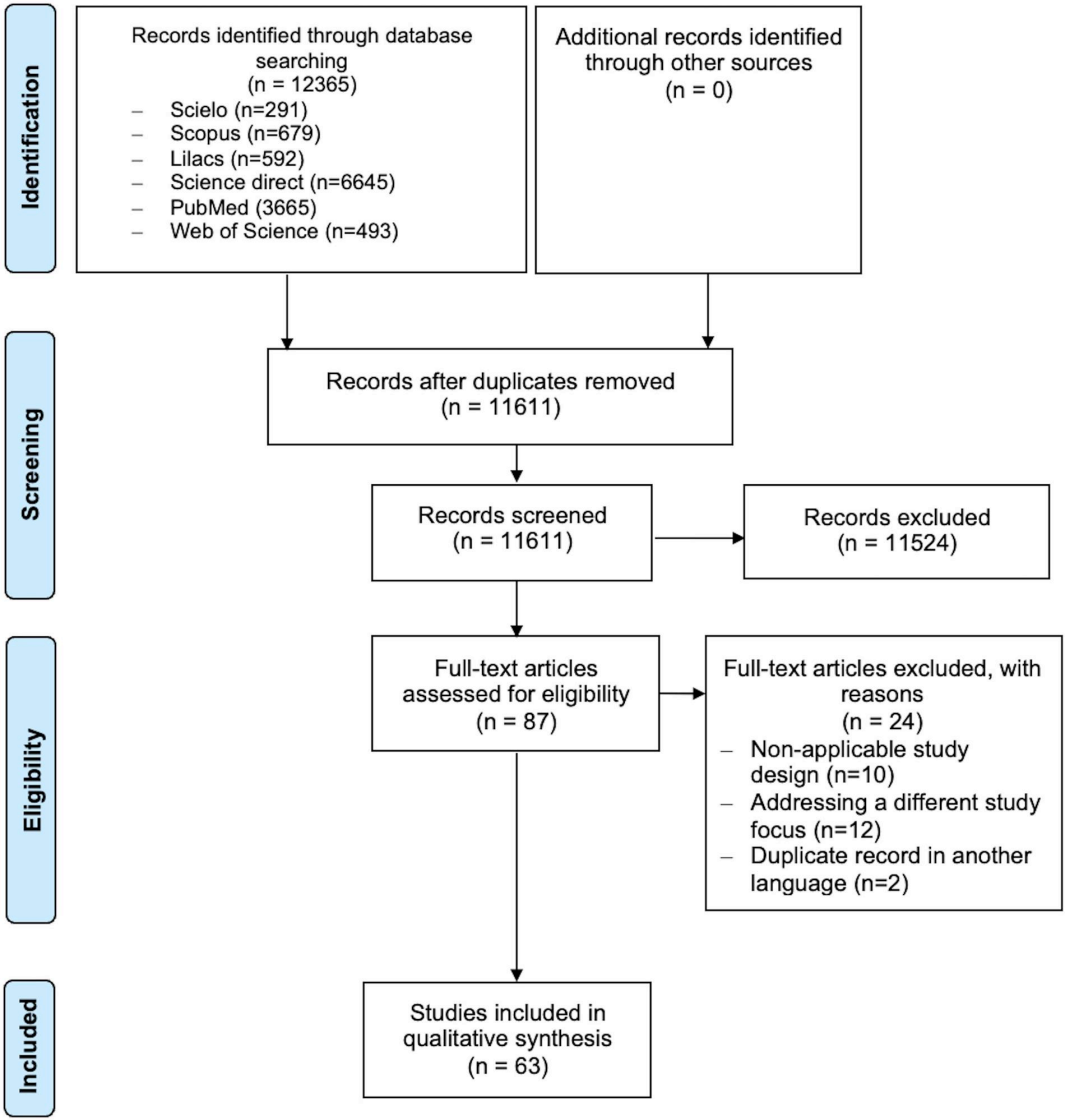
Literature search and selection. Diagram adapted from the flowchart for reporting systematic reviews or meta-analyses. PRISMA statement (16).

The review protocol was developed *a priori* and registered in PROSPERO under the registration number CRD42021262710. The study selection process involved a systematic screening of titles, abstracts, and full texts based on predefined eligibility criteria. Records were excluded due to non-applicable study design, lack of alignment with the research objective, or duplication in another language. The full selection process, including reasons for exclusion at each stage, is presented in the flow diagram (Figure 1).

The inclusion criteria comprised empirical studies addressing user's experiences with healthcare services and reporting findings related to any of the 14 guiding principles and/or the four essential elements of the FRH (3). Exclusion criteria included non-empirical publications (e.g., reflection or opinion papers), studies conducted outside the Colombian context, articles written in languages other than English or Spanish, and those not explicitly related to the principles and essential elements established by Law No. 1751 of 2015.

A total of 12,365 records were identified through the database search, of which 754 were removed as duplicates (15). The remaining 11,611 records were screened based on titles and abstracts to assess eligibility. Subsequently, 11,524 records were excluded due to non-relevant scope, language restrictions, or contextual mismatch. A total of 87 articles were retrieved for full-text review, of which 63 met the inclusion criteria and were included in the final analysis (Figure 1).

Data processing and analysis were conducted in three phases, following the methodological framework proposed by Whittemore and Knafl (14), which conceptualizes integrative review (IR) analysis as a process involving data reduction, data display, data comparison, and verification.

A qualitative approach based on content analysis was employed due to its capacity to systematically identify, classify, and interpret patterns within textual data, thereby transforming heterogeneous evidence into structured analytical categories (17, 18). This method is particularly well suited to integrative reviews, as it enables the synthesis of findings from diverse study designs while preserving their analytical depth (14).

A combined deductive-inductive coding strategy was applied. In the initial stage, text fragments were deductively mapped onto the predefined conceptual framework of the FRH. This was followed by an inductive coding phase, in which conceptually similar codes were grouped into first-order categories through constant comparison and thematic clustering (19).

The analytical process followed a hierarchical and iterative content analysis, in which categories were progressively refined and consolidated into higher-order analytical constructs. This reduction-from text fragments to codes, and from codes to inductive and broader conceptual categories-was conducted in accordance with established qualitative methodologies, ensuring coherence with the theoretical framework underpinning the study.

In the initial phase, data reduction was performed through the systematic coding of text fragments extracted from the selected studies. Analytical categories were developed based on semantic meaning and their alignment with the principles and essential elements of FRH, as established in Law No. 1751 of 2015. To enhance reliability and validity, a triangulation process was applied (20), involving the comparison of multiple sources of evidence, analytical tools, and theoretical references. Specifically, coded text fragments were cross-checked across studies, validated against the normative framework, and examined using complementary analytical matrices.

**Figure 2:**
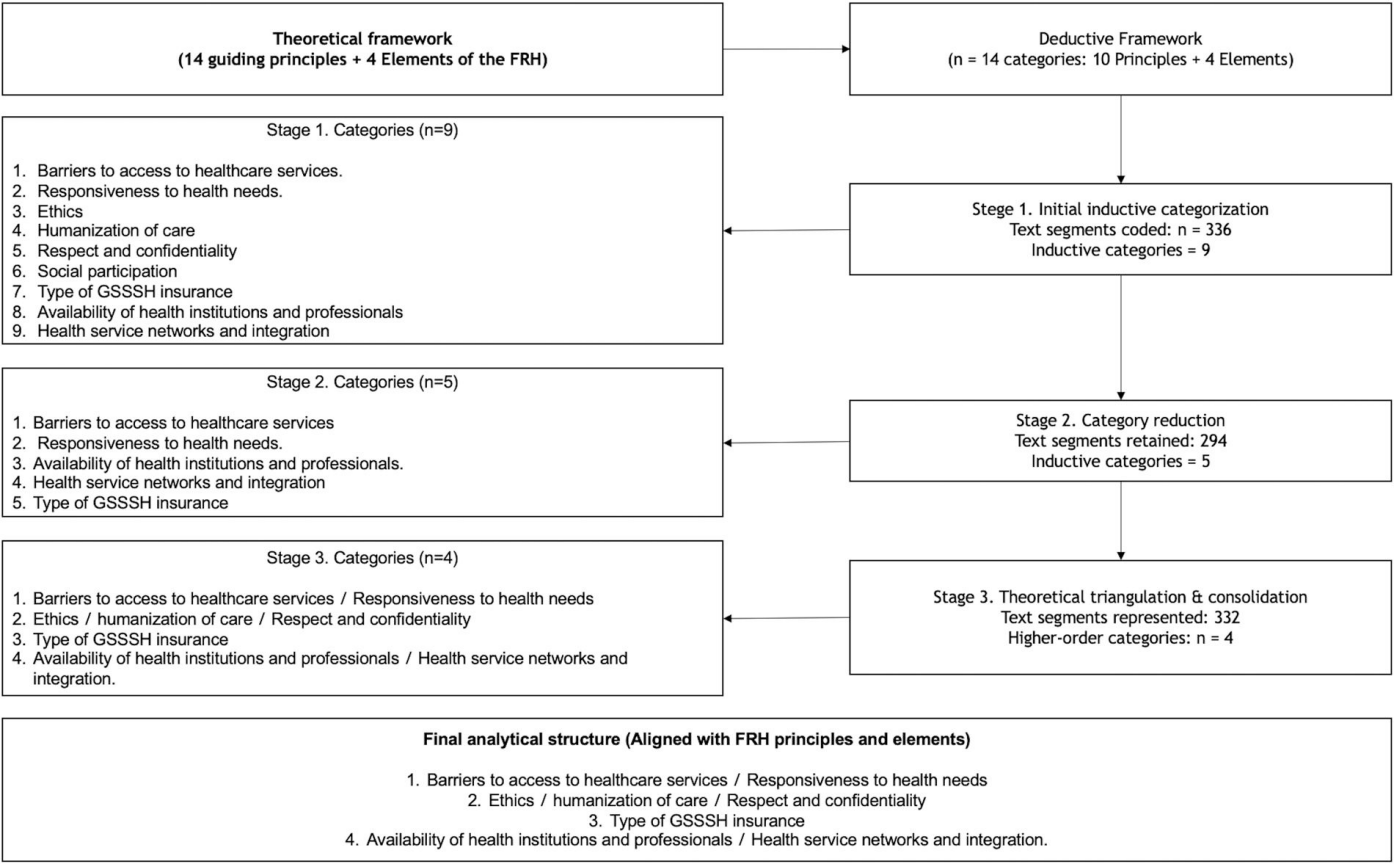
Multi-stage category development and reduction process. Prepared by the authors.

In the subsequent phase, categories were refined through an iterative process of comparison, abstraction, and thematic clustering, enabling the consolidation of conceptually related data into higher-order analytical categories. This systematic triangulation and iterative validation process ensured consistency in coding, strengthened the credibility of the findings, and improved the robustness of category development. The overall analytical process is illustrated in Figure 2 and supports the identification of key analytical variables relevant to subsequent empirical research in rural contexts.

Data extraction was carried out using a structured bibliographic matrix implemented in Rayyan (15), which included variables such as reference information, abstract, study design, data collection methods, study population, health condition or event examined, and study setting. This matrix was complemented by a second analytical tool developed using QDA Miner Lite v2.0.8 (Provalis Research), which facilitated the coding, organization, and analysis of text fragments (Supplementary Material 2). The coded data were subsequently validated through an affinity matrix (Supplementary Material 3), which enabled a systematic comparison of text fragments against each of the 14 principles and four essential elements of the FRH.

For analytical purposes, selected principles were either consolidated or excluded. The principles of “interculturality”, “protection of Indigenous peoples”, and “protection of Indigenous, ROM, Black, Afro-Colombian, Raizal, and Palenquero communities” were consolidated under a single interculturality dimension.

Conversely, the principles of “free choice” and “solidarity” were excluded due to the absence of supporting evidence in the analyzed text fragments. As a result, the original set of fourteen principles was reduced to ten. When combined with the four essential elements, this yielded a total of fourteen final analytical dimensions, as presented in Figure 2.

A total of 336 text fragments were extracted from the 63 included studies and subjected to a systematic coding process. Each fragment was independently coded and deductively mapped to the predefined framework of the FRH, with 150 fragments associated with the essential elements and 186 with the guiding principles.

Following this initial coding stage, an inductive analytical process was applied to group conceptually similar codes into higher-order categories. Through iterative comparison and thematic clustering, nine inductive categories were generated. The most frequently represented categories were “barriers to access to healthcare services” (*n* = 118), followed by “responsiveness to health needs” (*n* = 86), “type of SGSSS insurance” (*n* = 37), “availability of health institutions and professionals” (*n* = 33), “health service networks and integration” (*n* = 20), “ethics” (*n* = 15), “humanization of care” (*n* = 12), “respect and confidentiality” (*n* = 11), and “social participation” (*n* = 4). This coding and categorization process ensured a systematic linkage between empirical evidence and the theoretical framework, enhancing the analytical rigor and transparency of the findings.

The category development process followed a multi-stage analytical progression. In the initial stage, nine inductive categories were generated, representing the ten guiding principles and four essential elements of FRH. In the second stage, four categories with the lowest frequency of text fragments were excluded. In the third stage, a process of theoretical triangulation was applied, whereby previously excluded categories were reintegrated based on shared conceptual and theoretical linkages, as presented in Figure 2. This step increased the representativeness of the analyzed text fragments, thereby enhancing analytical clarity and ensuring alignment with the principles and essential elements identified in the reviewed studies.

The reduction was guided by methodological criteria aimed at maximizing explanatory power while minimizing the number of categories. This approach facilitated the progressive consolidation and refinement of categories, resulting in a more parsimonious and conceptually coherent representation of the findings.

Table 2 presents the progression of category reduction up to the third stage, along with their alignment with the principles and essential elements of the FRH. This framework supports the organization and presentation of the results, as well as the subsequent analysis and interpretation.

**Table 2:** Reduction of emergent categories and their alignment with the principles and essential elements of the FRH.

Final analytical categories (*n* = 4)	Inductive categories (source)	FRH mapping (summary)
Barriers to access to healthcare services/Responsiveness to health needs	Barriers to access to healthcare services; Responsiveness to health needs	Elements (*n* = 4): A, Ac, Ap & Q Principles (*n* = 10): Co, E, Eq, I, T, Pr, Pg, Ph, S, & U
Ethics/Humanization of care/Respect and confidentiality	Ethics; Humanization of care; Respect and confidentiality	Elements (*n* = 3): A, Ap & Q Principles (*n* = 6): E, Eq, I, T, Pg & Ph
Social participation: This category may be associated with both access barriers and ethical dimensions; however, it was represented by the lowest number of coded text fragments among all categories.		
Type of SGSSS insurance	Type of SGSSS insurance	Elements (*n* = 3): Ac, Ap & Q Principles (*n* = 4): Co, Eq, T & U
Availability of health institutions and professionals/Health service networks and integration	Availability of health institutions and professionals; Health service networks and integration	Elements (*n* = 3): Ac, Ap & Q Principles (*n* = 9): Co, E, Eq, I, T, Pr, Pg, S, & U

Elements (AAAQ): A, availability; Ac, accessibility; Ap, acceptability; Q, quality & professional competence.

Principles (CEEITPPPSU): Co, continuity; E, efficiency; Eq, equity; I, interculturality; T, timeliness; Pr, the primacy of rights; Pg, the progressivity of rights; Ph, pro homine; S, sustainability, & U, universality.

## Results

Principles and Essential Elements of the FRH: What Has Been Documented in Colombia?

Green and yellow areas represent regions with higher levels of scientific production on health system–related topics, whereas red and gray areas indicate regions with limited or no available publications, respectively. Within this framework, the evidence included in this review predominantly derives from studies conducted in Bogotá, Antioquia, and Valle del Cauca.

**Figure 3:**
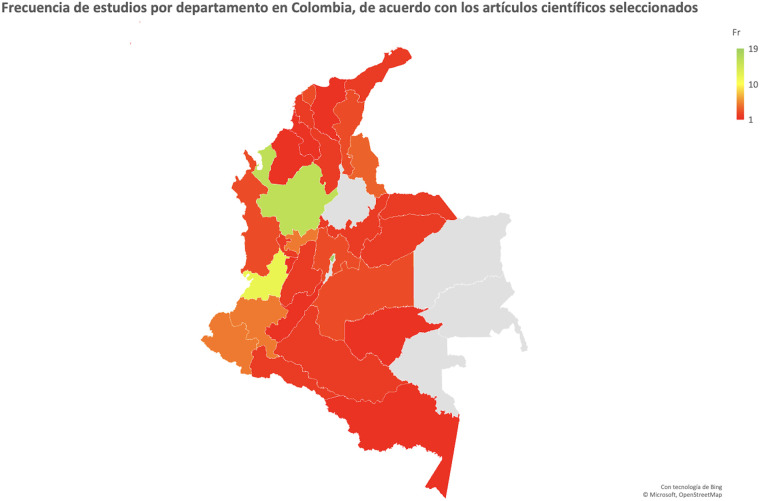
Reduction map detailing the frequency of studies of the 63 selected articles, by department, in Colombia. Own data generated using Bing technology. Microsoft, OpenStreetMap.

Although fewer studies were identified in regions such as Amazonas, Córdoba, Guaviare, Huila, Magdalena, Quindío, San Andrés, and Sucre, no studies meeting the inclusion criteria were identified for Santander, Guainía, Vaupés, and Vichada. These geographic patterns are illustrated in Figure 3.

The integrative review identified a higher concentration of eligible studies published between 2018 and 2020. Among the essential elements, accessibility, quality and professional competence, and availability were the most frequently represented in the coded text fragments, while equity, timeliness, continuity, and universality emerged as the dominant guiding principles. These patterns indicate that the most frequently coded elements and principles provide a robust representation of the core analytical categories (Table 3).

**Table 3:** Distribution of coded text fragments across essential elements and principles of the FRH.

FRH dimension	Component	Text fragments (*n*)	%
Essential elements of the FRH	Accessibility	139	42
	Quality and-professional competence	89	27
	Availability	59	18
	Acceptability	41	13
S/Total	–	328	100
Principles of the General Social Security Health System (FRH)	Equity	121	27
	Timeliness	96	22
	Continuity	68	15
	Universality	42	10
	Efficiency	27	6
	Primacy of rights	26	6
	Pro Homine	18	4
	Progressivity of rights	17	4
	Interculturality	16	4
	Sustainability	10	2
S/Total	–	441	100
TOTAL	–	769	–

Primary findings across the four higher-order categories are presented below:

1. Barriers to access to healthcare services/Responsiveness to health needs.

This category captured evidence of violations of the right to access healthcare, particularly among children (21–23), pregnant women (24, 25), older adults (26), adult men deprived of liberty (27), and individuals undergoing reintegration following the peace agreement (28). It also included Indigenous and Afro-descendant populations (29–31), many of whom live in rural, agricultural settings and experience socioeconomic constraints that constitute an additional barrier to access (24, 32–36). Among some ethnic communities, social participation was constrained by language barriers, which contributed to discriminatory practices and a limited relationship between communities and state institutions (37).

With respect to people with disabilities, the reviewed studies reported multiple access barriers, including denial or delays in obtaining medicines and assistive devices, delays or cancellations of authorizations for care and rehabilitation services, and limited access to specialized services due to the scarcity of institutions capable of providing the required level of complexity. Collectively, these factors contributed to fragmented care pathways (38–40). Studies involving this population were conducted in vulnerable areas of Bogotá, D.C. (21, 41), frequently among individuals affiliated with the subsidized insurance regime, and in other cases among residents of rural and geographically dispersed areas.

Overall, this category encompassed administrative and operational features of the SGSSS that adversely affected effective access, timeliness, and continuity across healthcare services. In addition, barriers to meaningful participation were identified, including insufficient training for members of associations or committees, political influence over these groups, weak oversight by control entities, unclear role definitions, and restricted access to information—factors that limit problem identification, corrective action, and the monitoring of healthcare delivery. These issues were also reported in user associations linked to EPS/EAPB structures (42).

2. Ethics/Humanization of care/Respect and confidentiality.

This category encompassed issues related to ethical breaches and the dehumanization of care, including insufficient provision of information and the failure to obtain informed consent for invasive procedures. The reviewed studies reported cases of surgical interventions performed outside established clinical protocols and scientific evidence, as well as instances of verbal abuse, coercion, abuse of authority, and hierarchical subordination within the doctor–patient relationship. Additionally, a lack of sensitivity to cultural differences in healthcare practices was identified (24, 35, 41, 43–47).

Furthermore, inappropriate communication was reported, particularly in interactions with older adults, Indigenous, and Afro-descendant patients, where responses did not adequately reflect their needs or cultural contexts. The dehumanization of care was also linked to institutional constraints that limited medical autonomy and imposed rigid service schedules at the expense of patients' needs (48).

Unequal treatment of individuals from vulnerable and minority groups was frequently described, including pregnant women, people with disabilities, and older adults. These disparities contributed to the deterioration of health conditions and reinforced existing inequities in access to care and quality of services (46, 48).

3. Type of SGSSS insurance.

This category examines the role of insurance affiliation within the SGSSS as a determinant of differential access to healthcare services. The evidence suggests that access is conditioned by the type of insurance regime, disproportionately affecting vulnerable populations and reinforcing inequities in health outcomes (25, 27, 29, 31, 33–35, 47, 49–56).

4. Availability of health institutions and professionals/Health service networks and integration.

This category addresses structural barriers related to the organization and distribution of healthcare providers within the system. The findings highlight the unequal geographical distribution of healthcare institutions (IPS), particularly those providing secondary and tertiary care (levels II and III), as well as specialized services such as laboratories and referral centers. These services are predominantly concentrated in major urban areas, where population density and economic flows are higher, resulting in better availability of specialized human resources, as well as technical and technological capacity (21, 24, 25, 32, 34, 36, 57–59).

The reviewed studies also emphasized challenges in the functioning of referral and counter-referral systems, which are essential for ensuring accessibility, continuity, and quality of care. These challenges include delays associated with geographic distance, insufficient consideration of patients' cultural contexts, limited recognition of alternative health practices, and the persistence of discriminatory practices within service delivery. Collectively, these factors contribute to inefficiencies in care coordination and hinder timely access to appropriate services (24, 34, 36, 40, 58, 59).

Furthermore, these findings reflect broader limitations in health system integration, particularly regarding the coordination between providers and insurers. The evidence indicates that administrative and financial priorities often prevail over patient-centered care, in contrast to the principles established in Law No. 1751 of 2015 (21, 24, 32, 34, 47). Table 4 summarizes the distribution of principles and essential elements identified in the integrative review.

**Table 4:** Analytical categories, conceptual basis, and key empirical findings related to the fundamental right to health (FRH).

Final category	Conceptual basis (FRH dimension)	Core analytical interpretation	Key empirical findings	References
Barriers to access to healthcare services/Responsiveness to health needs	Essential elements (Q, A, Ap) + Principles (Eq, S)	Violations of the right to health driven by administrative, operational, and structural barriers affecting vulnerable populations.	Delays in care; insufficient human resources and technologies; geographical inequalities; weak oversight; limited user participation due to lack of training, political influence, and limited access to information.	(22, 25, 26, 28, 31, 35–39, 41, 42, 52)
Ethics/Humanization of care/Respect and confidentiality	Essential elements (Q, Ap) + Principles (Ph, I)	Ethical failures and institutional constraints contributing to the dehumanization of care and unequal treatment.	Lack of informed consent; verbal abuse; coercion; limited cultural sensitivity; restricted medical autonomy; inadequate communication with vulnerable groups; disparities in care affecting pregnant women, older adults, and people with disabilities.	(35, 40, 41, 43, 44, 46, 72–74)
Type of SGSSS insurance	Essential elements (Ac) + Principles (Eq, T, Pg, U)	Differential access to healthcare services conditioned by insurance affiliation, reinforcing inequities.	Barriers in subsidized regimes; fragmented care; delays in treatment; higher burden among vulnerable groups (Afro-descendants, Indigenous populations, displaced individuals, elderly populations).	(24, 33, 50, 57, 65–67)
Availability of health institutions and professionals/Health service networks and integration	Essential elements (A, Ac, Q) + Principles (Co, U, E, Pr, Pg, I)	Territorial and systemic inequities due to uneven distribution of services and weak network integration.	Concentration of specialized services in urban areas; limited referral systems; delayed care; lack of cultural adaptation; fragmented service delivery; prioritization of financial/administrative efficiency over patient-centered care.	(21, 24, 25, 30, 32, 34, 36, 45, 46, 57–59, 65, 73, 75)

Principles (CEEITPPPSU): Co, continuity; E, efficiency; Eq, equity; I, interculturality; T, timeliness; Pr, the primacy of rights; Pg, the progressivity of rights; Ph, pro homine; S, sustainability, & U, universality.

Elements (AAAQ): A, availability; Ac, accessibility; Ap, acceptability; Q, quality & professional competence.

Taken together, the findings reveal a convergence of access barriers, ethical challenges, systemic fragmentation, and insurance-based inequalities, all of which interact to influence the effective realization of the FRH.

The Discussion section builds on these results by situating them within the broader literature on health system governance and social determinants of health, and by examining their implications for policy, practice, and future research.

## Discussion

Taken together, the four analytical categories demonstrate that the realization of the FRH in Colombia—before, during, and after the enactment of Law No. 1751—has been consistently challenged by structural determinants. These factors contribute to persistent health inequities, manifested through barriers to accessing healthcare services, the dehumanization of care, discrimination related to insurance affiliation (subsidized versus contributory regimes), and unequal availability of health institutions, services, and professionals, particularly in vulnerable, rural, and geographically dispersed areas.

According to the literature review, the exercise of the principles and essential elements of the FRH in Colombia is closely aligned with the structural determinants of health inequities (12). This pattern is consistent with both prior and subsequent analyses conducted in the country (9, 10), which emphasize the persistence of systemic barriers within the health system.

Within this context, the most salient issues identified were related to access, availability, and quality of health services, with pronounced territorial disparities, particularly in rural and dispersed areas. These disparities reflect structural inequities associated with institutional capacity for service provision and the uneven distribution of healthcare professionals (60).

Primary healthcare (PHC) in Colombia has likewise been constrained by these structural determinants, falling short of its expected development. Persistent barriers include ethical tensions between institutional efficiency and the primacy of rights, weaknesses in service networks and their integration, and comparable deficiencies across both subsidized and contributory regimes. Collectively, these constraints hinder the delivery of comprehensive and integrated care, encompassing public health, health promotion, disease prevention, diagnosis, treatment, and rehabilitation across all levels of complexity (61).

Failures in governance and regulatory control were also evident across the reviewed studies, particularly in the implementation of public policies by the governing body and other actors responsible for administering, regulating, monitoring, and ensuring accountability within the system. These combined weaknesses limit the capacity of the health system to ensure equitable and high-quality access to care (22, 41).

Moreover, persistent delays in service delivery, limited access to medical technologies and supplies, and weaknesses in health system monitoring—even in urban settings—reflect administrative inefficiencies and reduced system resilience within the SGSSS, thereby constraining the effective realization of the right to health (25, 33, 34, 42, 62, 63).

In line with international literature, strengthening governance and regulatory mechanisms is essential to addressing these systemic challenges, as they are critical determinants of health system performance (11, 64).

Furthermore, the findings suggest that the insurance-based model functions as a key driver of structural inequities. This dynamic is influenced by the country's economic model, as well as the prevailing social and political priorities of decision-makers, which often take precedence over population health needs. Consequently, access to healthcare services remains conditioned by the type of insurance affiliation, disproportionately affecting individuals under the subsidized regime (30, 31).

As observed in this review, insurance-based segmentation contributes to fragmented care pathways, delays in treatment, and reduced continuity of care, disproportionately undermining the health of vulnerable populations such as Indigenous groups, Afro-descendants, displaced persons, and older adults (24, 33, 50, 57, 65–67).

From a theoretical perspective, these findings align with critical analyses of health system segmentation in Latin America, which demonstrate that insurance-based models tend to reproduce social inequities through stratification mechanisms (68, 69).

When interpreted through the principles and essential elements of the FRH, and considering the four analytical categories identified in this review, the findings reveal a scenario in which the normative objectives of the right are not fully achieved. However, this challenge is not unique to Colombia. For example, evidence from health system and public health reforms in Greece indicates that policy implementation alone is insufficient to address the demands and complexities of contemporary health systems.

In that context, structural determinants have similarly contributed to the fragmentation and weakening of service delivery, affecting both quality and responsiveness to population needs (70). Despite differences between countries in economic capacity, demographic composition, and sociocultural characteristics, common challenges persist, including weak regulation, insufficient monitoring, and system inefficiencies that ultimately lead to increased out-of-pocket expenditures.

Across the four analytical categories examined in this study, parallels with the Greek case can be observed, particularly in relation to barriers to access, the dehumanization of care, and the limited availability of health institutions, services, and professionals.

Although the findings predominantly indicate systemic limitations, some positive experiences were identified in specific contexts, such as the post-implementation evaluation of peace agreement programs (28), where improvements were observed in timeliness, quality of care, and access to medicines. However, these cases appear to be exceptional, as they are associated with targeted investments and specific funding schemes rather than reflecting structural improvements in the health system.

The predominance of systemic limitations, coupled with limited successful experiences in strengthening the fundamental right to health, is consistent with global trends. The World Health Organization has reported a decline in financial support for health systems in low- and middle-income countries between 2015 and 2023, which has negatively impacted the recruitment of healthcare personnel. This situation has been exacerbated by the effects of the COVID-19 pandemic, which exposed and intensified structural weaknesses, ultimately affecting progress toward universal health coverage (71).

At a global level—like the Colombian case—these dynamics negatively affect the four analytical categories identified in this review. Based on these findings, further research is needed to deepen the understanding of access barriers, the processes leading to dehumanization of care, the role of discrimination associated with insurance regimes (subsidized versus contributory), the dynamics between public and private healthcare provision, and the availability of services and healthcare professionals.

Such efforts are essential to inform governance strategies aimed at addressing these challenges and strengthening the structural foundations of the health system, ultimately contributing to the effective realization of the principles and essential elements of the FRH.

Limitations and strengths. This study has several limitations that should be considered when interpreting the findings. Although the integrative review (IR) sought to provide a comprehensive synthesis and followed key elements of PRISMA guidelines, it remains subject to inherent methodological constraints, including potential bias and interpretative subjectivity.

First, selection bias may have influenced the results, as the analysis focused exclusively on empirical studies, potentially omitting relevant theoretical or policy-oriented contributions. Second, language bias may be present, given that only studies published in English and Spanish were included.

Furthermore, the analytical process itself—particularly coding, categorization, and theoretical triangulation—may introduce interpretative bias, despite efforts to ensure methodological rigor. Accordingly, the findings should be interpreted with caution as indicative rather than generalizable, underscoring the need for further applied and context-specific research to validate and expand these results.

Despite these limitations, this study also presents important strengths. The integrative review design enabled the inclusion and systematic synthesis of diverse types of evidence, providing a comprehensive perspective on the implementation of the fundamental right to health in Colombia. The use of a combined deductive–inductive analytical approach, supported by theoretical triangulation, enhanced the robustness and coherence of category development.

Additionally, the explicit integration of a rights-based framework with governance and social determinants perspectives offers a novel analytical lens for understanding structural inequities. Finally, the identification of higher-order analytical categories contributes a structured foundation for future empirical research and policy development, particularly in contexts characterized by territorial disparities and vulnerable populations.

## Conclusions

The findings of this integrative review demonstrate that the realization of the fundamental right to health in Colombia remains constrained by structural, institutional, and organizational factors.

Key challenges include persistent barriers to access, ethical limitations in care delivery, inequities associated with insurance affiliation, and territorial disparities in service availability. These challenges disproportionately affect vulnerable populations.

The four analytical categories identified provide a useful framework for future research and policy development. However, further empirical studies are required to validate these findings and explore context-specific solutions.

Overall, addressing these challenges requires a comprehensive approach focused on strengthening governance, reducing system fragmentation, and promoting equity in healthcare delivery.

## Supplementary material

Supplementary Material 1 Search plan applied.

Supplementary Material 2 A table through which text fragments were consolidated and analyzed, generated by PROVALIS' QDA Miner Lite v2.0.8.

Supplementary Material 3 Validation by means of an Affinity Matrix with each of the principles and essential elements of the FRH from the definitions expressed in statutory law 1751 of 2015.
